# A moderate increase in dietary zinc reduces DNA strand breaks in leukocytes and alters plasma proteins without changing plasma zinc concentrations^1^,^2^,^3^

**DOI:** 10.3945/ajcn.116.135327

**Published:** 2016-12-21

**Authors:** Sarah J Zyba, Swapna V Shenvi, David W Killilea, Tai C Holland, Elijah Kim, Adrian Moy, Barbara Sutherland, Virginia Gildengorin, Mark K Shigenaga, Janet C King

**Affiliations:** Nutrition and Metabolism Center, Children’s Hospital Oakland Research Institute, Oakland, CA

**Keywords:** antioxidant, DNA repair, inflammation, zinc biomarkers, zinc fortification

## Abstract

**Background:** Food fortification has been recommended to improve a population’s micronutrient status. Biofortification techniques modestly elevate the zinc content of cereals, but few studies have reported a positive impact on functional indicators of zinc status.

**Objective:** We determined the impact of a modest increase in dietary zinc that was similar to that provided by biofortification programs on whole-body and cellular indicators of zinc status.

**Design:** Eighteen men participated in a 6-wk controlled consumption study of a low-zinc, rice-based diet. The diet contained 6 mg Zn/d for 2 wk and was followed by 10 mg Zn/d for 4 wk. To reduce zinc absorption, phytate was added to the diet during the initial period. Indicators of zinc homeostasis, including total absorbed zinc (TAZ), the exchangeable zinc pool (EZP), plasma and cellular zinc concentrations, zinc transporter gene expression, and other metabolic indicators (i.e., DNA damage, inflammation, and oxidative stress), were measured before and after each dietary-zinc period.

**Results:** TAZ increased with increased dietary zinc, but plasma zinc concentrations and EZP size were unchanged. Erythrocyte and leukocyte zinc concentrations and zinc transporter expressions were not altered. However, leukocyte DNA strand breaks decreased with increased dietary zinc, and the level of proteins involved in DNA repair and antioxidant and immune functions were restored after the dietary-zinc increase.

**Conclusions:** A moderate 4-mg/d increase in dietary zinc, similar to that which would be expected from zinc-biofortified crops, improves zinc absorption but does not alter plasma zinc. The repair of DNA strand breaks improves, as do serum protein concentrations that are associated with the DNA repair process. This trial was registered at clinicaltrials.gov as NCT02861352.

## INTRODUCTION

The dependence on cereal-based diets in lower-income countries has led to the development of fortification programs to enhance the micronutrient amounts of staple food crops including rice, wheat, and maize. Because of the need for adequate zinc intakes to support optimal child health and growth and for normal pregnancy outcomes, >30 countries have initiated zinc-fortification programs (1). In addition to adding zinc to various cereal flours postharvest, biofortification methods (i.e., selective breeding and the use of zinc-containing fertilizers) have been used to increase the zinc concentrations in crops (2, 3). For example, Bangladeshi researchers increased the rice zinc concentration by 50%, from 16 to 24 parts per million (ppm)^5^, through biofortification methods (4). In populations who derive ∼80% of their total energy intake from rice, as is the case in very poor households, a 50% increase in the rice zinc concentration would translate to an additional ∼4 mg Zn/d in the total diet. Previous studies have shown that, when infants or children are fed zinc-fortified cereals, the additional zinc that is provided by the crop ranges from only 1.5 to 3 mg/d (5). Although zinc fortification is a safe strategy for enhancing zinc intakes of populations, none of the cereal-based zinc-fortification programs had a positive effect on child growth or morbidity.

In addition to influencing growth and morbidity, zinc attenuates oxidant stress over the range of usual zinc intakes, and in that capacity, zinc modulates DNA damage (6, 7). Increased zinc availability reduces the amounts of reactive oxygen species (ROS) and the concentrations of inflammatory cytokines and chemokines (8–10). An attack by ROS is one of the primary endogenous causes of the thousands of lesions of cellular DNA per day (11). However, modest changes in dietary zinc can modulate DNA damage primarily by reducing cellular oxidative stress (7). In a study of adult men, we showed that leukocytic DNA strand breaks increased when only 4 mg Zn/d was consumed, and the number of breaks declined when dietary zinc was increased to 11 mg/d (12). This association between zinc, oxidative stress, and inflammation may explain the association between zinc and chronic disease (13).

To our knowledge, the effect of modest increases in dietary zinc on DNA damage, as would occur when zinc-fortified cereals are added to the diet, has not been previously studied. We hypothesized that an increase in cellular oxidative stress and DNA damage is one of the earlier signs of reductions in cellular zinc (7, 14); therefore, a modest increase in dietary zinc would reduce DNA damage and alter the concentrations of serum proteins that are involved in DNA repair, oxidative stress, and inflammation. We also hypothesized that this modest 4-mg increase in dietary zinc would not increase plasma zinc concentrations (PZCs) or the size of the exchangeable zinc pool (EZP). If confirmed, these findings would provide a zinc functional biomarker that is sensitive to modest physiologic changes in dietary zinc.

## METHODS

### Study design

Eighteen healthy men were recruited from March 2012 to July 2013 to participate in a nonblinded, randomized, controlled, 6-wk zinc depletion and repletion study. After a 3-d run-in period, the men consumed a controlled diet that provided 6 mg Zn/d for 2 wk [metabolic period 1 (MP1)]. A total of 1.5 g sodium phytate, which would bring the diet phytate:zinc molar ratio to 44, was distributed evenly into 3 meals to facilitate zinc depletion by limiting zinc absorption. Thereafter, dietary zinc was increased to 10 mg/d by adding a total of 4 mg exogenous zinc (i.e., 1.33 mg Zn/meal) into the rice portion of the meal for the remaining 4 wk. The rice was cooked in a conventional rice cooker according to the manufacturer’s instructions at 360 mL H_2_O/240 mL rice for 30 min or until all free water was absorbed. Rice was allowed to cool before the application of a sterile solution of 1 mg zinc chloride/mL directly to the rice. The rice was allowed to fully absorb the zinc solution before packaging. Portions of each batch of rice were also taken for a zinc analysis. In this second metabolic period, the additional phytate was discontinued. The men were not confined to a metabolic unit, but they came to the clinical site to have their vital signs checked and to pick up their food 2 times/wk. Fasting blood samples were collected at the beginning of the study (day 1) and at the end of MP1 and metabolic period 2 (MP2) (i.e., days 15 and 43).

### Ethics

The Children’s Hospital Oakland Research Institute Institutional Review Board approved the study, and written informed consent was obtained from each participant.

### Participants

Healthy adult men, who were aged 19–45 y with BMI (in kg/m^2^) between 18 and 30, participated in the study. Before the study, the men discontinued any dietary supplements that contained zinc for ≥4 wk. Inclusion criteria consisted of a baseline PZC >60 μg/dL and usual zinc intake, as estimated from a 24-h dietary recall, of >9.5 mg Zn/d. Exclusion criteria included a self-report of chronic or acute diseases (including diabetes or asthma), the use of medications for a metabolic disorder (including statins or diuretics), and evidence of alcohol abuse or use of illicit drugs. Participant height was measured at the first clinical visit, and body weight was measured 2 times/wk during the study. Body composition was measured at the beginning of the study and at the end of MP2 (i.e., days 1 and 43) with the use of a BOD POD whole-body air-displacement plethysmography device (COSMED USA).

### Study diets

The study diet consisted of a 4-d cycle menu with 80% of energy from carbohydrate, 10% of energy from protein, and 10% of energy from fat. A description of the menus is shown in **Supplemental Table 1**. The macronutrient composition of the study diet was determined with the use of the University of Minnesota Nutrition Data System for Research 2010 (Nutrition Coordinating Center, University of Minnesota). Participants were permitted to add salt and pepper to the meals and to consume tap water ad libitum. Consumption of alcoholic drinks, soda, and other beverages were not allowed. Participants came to the clinical site biweekly to return food containers and to pick up food for the next 3–4 d. Any uneaten food was recorded at this time. The study coordinator also regularly observed food consumption in person or by video conferencing, which was scheduled during meal times via the Google Hangout platform (Google Inc.).

Energy requirements of the volunteers were estimated with the use of the resting metabolic rates that were calculated with the use of the BOD POD device at the start of the study and self-reported physical activity levels. Individual adjustments in dietary energy to maintain body weight were made by adding 1 of 2 zinc-free beverages with the same distribution of energy sources as in the overall study diet (Supplemental Table 1). Total energy intakes ranged from 2500 to 3000 kcal/d.

A macronutrient and micronutrient analysis was performed on duplicate portions of all meals for each of the 4 d in a cycle. The meals were homogenized and blended into whole-day composites. An additional complete meal was saved from each meal-preparation batch. Food and beverages were combined in a large commercial blender until fully liquefied. Only the blender blades were metal, and there was no obvious corrosion damage. The composite samples were batched at −20°C. Mineral concentrations, including zinc, were analyzed with the use of inductively coupled plasma optical emission spectrophotometry (ICP-OES) (Varian Vista Pro). The ICP-OES was calibrated with the use National Institute of Standards and Technology (NIST)–traceable elemental standards and routinely validated with the use of NIST-traceable 1577b bovine-liver reference material and Seronorm Trace Element Serum Levels 1 and 2 (Sero AS). The mean daily mineral amounts of the diet were as follows: 6.4 ± 1.5 mg Zn, 7.9 ± 2.1 mg Fe, 1.6 ± 0.5 mg Cu, 414.5 ± 24.9 mg Ca, 824.6 ± 148.7 mg P, and 250.0 ± 34.5 mg Mg. Although some of these mineral intakes were below Recommended Dietary Allowances (15), supplements were not provided in this short-term study. Phytate concentrations were measured with the use of high-performance anion-exchange chromatography followed by conductivity detection (16).

### Blood sampling and processing

Fasting blood samples were drawn between 0700 and 0900 at the beginning of the study (day 1) and at the end of each metabolic period (days 15 and 43). Trace metal–free certified evacuated tubes containing K_2_EDTA (Becton-Dickson) were used for the zinc measurements, the complete blood count, DNA integrity, and plasma protein concentrations. PAXGene tubes (Becton-Dickson) were used to collect samples for gene-expression measurements. Blood in the K_2_EDTA evacuated tubes was centrifuged at 800 × *g* for 15 min at 4°C before removing the plasma, leukocytes, and erythrocytes. The plasma was further centrifuged at 3000 × *g* for 10 min at 4°C to remove any remaining leukocytes and was stored at −80°C. Isolated leukocytes were washed twice with 10 vol RBC Lysis Solution (0.15 M NH_4_Cl, 10 mM KHCO_3_, and 0.3 mM EDTA; pH = 7.5) to remove contaminating erythrocytes, and the leukocytes were pelleted by centrifugation (500 × *g* for 10 min at 4°C). Isolated erythrocytes and leukocytes were suspended in a Cell Wash Solution (50 mM Tris-HCl and 150 mM NaCl; pH = 7.4) and counted with the use of an automated complete blood count instrument (ADVIA 120 Hematology System; Siemens Healthcare GmbH) according to the manufacturer’s protocol. Predetermined amounts of erythrocytes and leukocytes were pelleted by centrifugation (500 × *g* for 10 min at 4°C), cleared of supernatant fluid, and frozen at −80°C for analysis. Whole blood was also run on the ADVIA 120 Hematology System to monitor standard hematologic variables.

### Elemental analysis

Composite food, plasma, and blood cell zinc concentrations were determined with the use of ICP-OES (17–20). Briefly, samples were fully dissolved in 0.25 mL OmniTrace 70% HNO_3_ (EMD Chemicals) by microwave digestion with the use of a MARS5 Microwave Digestion Oven (CEM) at 100°C for 15 min. Samples were diluted to 5% HNO_3_ with OmniTrace water (EMD Chemicals), clarified by centrifugation (3000 × *g* for 10 min), and analyzed with the use of a Vista Pro ICP-OES (Varian Vista Pro). The ICP-OES was calibrated with the use of NIST-traceable elemental standards and validated with the use of NIST-traceable 1577b bovine-liver reference material. Zinc was measured at the 213-nm wavelength with a detection range between 0.005 and 5 ppm and a CV for interassay precision <10%. Cesium (50 ppm) was used for ionization suppression and yttrium (5 ppm) was used as an internal standard for all samples. All reagents and plastic ware were certified or routinely tested for trace-metal work. Zinc concentrations were expressed per volume (plasma) or per cell number (erythrocytes and leukocytes). Plasma and erythrocytes samples were analyzed in triplicate, and leukocyte samples were analyzed in duplicate. All samples from each participant were analyzed in the same run.

### Stable-isotope preparation

Zinc stable isotopes ^67^Zn (93.1% enriched) and ^70^Zn (89.6% enriched) were purchased as zinc oxide salts (Oak Ridge National Laboratory). The zinc salts were dissolved in concentrated hydrochloric acid (4.5 μL HCl/mg ZnO) (Optima grade, A-466; Fisher Scientific), diluted with triply deionized water to a final concentration of 1.0 mg ^67^Zn/mL or 0.1 mg ^70^Zn/mL, and filtered through a 0.22-μM filter (Millipore). The zinc solutions were tested for sterility and pyrogenicity and stored in individual, sealed, sterile vials by the School of Pharmacy, University of California at San Francisco, for use by intravenous administration.

### Isotope administration and measurement

Stable isotopes were administered on days 4 and 18 for measuring fractional zinc absorption (FZA), total absorbed zinc (TAZ), and the EZP. ^70^Zn was also infused on day 46 for measuring the EZP after MP2. For the studies of zinc absorption on days 4 and 18, participants were fed the first day of the 4-d study-diet rotation. A total of 1 mg ^67^Zn was divided into 3 equal doses (∼0.33 mg) and added as a fortificant to each meal. Phytate was not added to the 3 study meals on the isotope administration day to prevent interference with the isotope measurements. One hour after the dinner meal, 0.1 mg ^70^Zn was infused into the antecubital vein over 1–2 min. The catheter tubing was flushed with 3 mL sterile saline. The exact amount of tracer infused was determined from the difference in syringe weight before and after the infusion. The first urinary void was collected for 5 d (days 3–7) after each isotope infusion for the measurement of zinc isotopic ratios.

### Stable-isotope analysis

Zinc isotopic ratios were analyzed in the urine samples with the use of inductively coupled plasma mass spectrometry as reported previously (18–20). Urinary macrominerals were removed by adjusting the sample pH to 5.3 and applying the sample to chelating resin (Chelex 100; Bio-Rad). The samples were purified with the use of ion-exchange chromatography (AG8; Bio-Rad), and pure zinc was eluted with 0.0005 N HCl, evaporated to dryness, and dissolved in 1% HNO_3_ for inductively coupled plasma mass spectrometry analysis. The tracer:tracee method (^67^Zn:^66^Zn and ^70^Zn: ^66^Zn) was used to estimate the FZA with the use of a modification of the double-isotope tracer method (21). TAZ was calculated by multiplying total the amount of dietary zinc per day by the FZA. The EZP was estimated with the use of modifications of the Miller method in which urine tracer:tracee ratios were graphed onto a semilog plot to extrapolate the size of the pool at the time of infusion with the use of the *y* intercept (22).

### DNA integrity

DNA single-strand breaks in peripheral blood cells were determined with the use of a comet assay as described by Singh et al. (23). Briefly, 500 μL fresh whole blood was cryopreserved in liquid nitrogen. Cryopreserved blood was thawed rapidly and centrifuged at 400 × *g* for 5 min. Cell pellets were mixed with 500 μL of 0.5% low–melting point agarose at ∼37°C and applied onto microscope slides (Trevigen). Slides were incubated in lysis buffer (Trevigen) for 1 h, and DNA was allowed to unwind in alkali buffer (0.3 M NaOH/L and 1 mmol/L EDTA) for 20 min. Slides were electrophoresed for 20 min at 300 mA and 15 V and neutralized with 0.4 M Tris/L (pH = 7.5). The slides were washed in cold 100% methanol and 100% ethanol for 5 min each and air dried for storage. Immediately before analysis, nuclear material was stained with 65 μL 1× SYBR Green (Life Technologies)/field. Comet measurements were made with the use of a Zeiss Axiovert fluorescent microscope (Zeiss) and Komet 6 software (Andor Technology). Images of 300 randomly selected nuclei (150 nuclei from each of 2 replicate slides) were analyzed from each sample. No identifying information was available to the laboratory technicians who performed the assay. The olive tail moment was used to indicate DNA damage. The intraindividual variation was minimized with the use of each individual as his own control and a paired-analysis approach. For detecting oxidized bases, a digestion step with a bacterial repair enzyme was included in the alkaline comet assay before electrophoresis in accordance with the modified protocols of Collins et al. (24). Briefly, after the lysis step, slides were washed 3 times in enzyme buffer (40 mmol HEPES/L, 100 mmol KCl/L, 0.5 mmol Na_2_EDTA/L, and 0.2 mg bovine serum albumin/mL; pH 8.0) and incubated with 100 mU formamidopyrimidine DNA-glycosylase (FPG) (New England Biolabs) for 30 min at 37°C. Amounts of oxidized sites were calculated from the olive tail moment score that was obtained with the enzyme minus the score that was obtained without the enzyme.

### Proteomic analysis

The SOMAscan proteomic assay (SomaLogic Inc.) was used to measure 1129 proteins in plasma samples that collected at the end of MP1 and MP2 (days 15 and 43). The assay has been described more extensively elsewhere (25) and on the SomaLogic Inc. website (http://www.somalogic.com/Products-Services/SOMAscan/FAQs.aspx). Proteins were measured with the use of a SOMAmer-based capture array (SomaLogic Inc.) SOMAmers are single-stranded DNA aptamers, which include some bases that have been chemically modified to mimic protein side chains while retaining the ability to pair with standard DNA bases [allowing their amounts to be quantified with the use of a polymerase chain reaction (PCR) or microarrays]. A total of 1129 of these SOMAmers are currently available, and they can be used to analyze a biological sample in such a way that the amount of each type of SOMAmer that remains in the sample is proportional to the amount of the corresponding protein targets in the original sample. Currently, one-third of SOMAmers have been assessed for specificity with the use of a pulldown and mass spectrometry. Proteomic data were collected with the use of a fluorescent imager (SOMAscan v3; SomaLogic Inc.) for the measurement of 1129 proteins. Quality control was performed at the sample and SOMAmer levels and involved the use of control SOMAmers on the microarray and calibration samples. At the sample level, hybridization controls on the microarray were used to monitor the sample-by-sample hybridization variability, whereas the median signal over all SOMAmers was used to monitor the overall technical variability. The resulting hybridization scale factor and median scale factor were used to normalize data across samples. Plasma samples from subjects at the 2 time points were randomly assigned to plates within the array and analyzed along with a set of calibration and normalization samples. No identifying information was available to the laboratory technicians who performed the assay. All samples passed quality-control criteria and were used for the analysis. The SOMAscan proteomic data are reported in relative fluorescence units. Relative fluorescence-unit data were log-transformed before the statistical analysis to reduce heteroscedasticity.

### Preparation of leukocyte total RNA and real-time PCR analysis of zinc transporter expression

Blood collected in PAXGene tubes was incubated at room temperature for 2 h and immediately frozen at −80°C until analysis. RNA extraction was completed with the use of a PAXgene Blood RNA kit (Qiagen) according to the manufacturer’s protocol. Total RNA was treated with DNase 1 to avoid any DNA contamination. The concentrations and purities of the extracted RNA samples were evaluated by measuring absorbance at 260 and 280 nm wavelengths with the use of a spectrophotometer (NanoDrop; Thermo); only samples with optical density 260:optical density 280 values ≥1.8 were used in the analysis. Complementary DNA was synthesized from 1 μg total RNA with the use of a QuantiTect RT kit (Qiagen) according to the manufacturer’s protocol. Relative messenger RNA (mRNA) concentrations of specific zinc transporter genes were measured with the use of a quantitative real-time PCR. All quantitative real-time PCR assays were conducted with SYBR Green dye (Qiagen) on an ABI 7900 HT system (Life Technologies). All primers were designed with the use of a Universal Probe Library System Assay Design (Roche) (**Supplemental Table 2**). The specificity of each primer pair was confirmed with the use of melting curve analyses, and the relative quantity was determined by normalization with respective 18S RNA and β-actin values with the use of the ΔΔ threshold cycle method.

### Sample-size calculations and statistical analysis

We planned to enroll 20 men, which, according to previous research, would have enabled us to determine a 5% difference in FZA within subjects between the 2 metabolic periods with α = 0.05 and β = 0.20 (20, 26). On the basis of a sample of 18 men and the observed variation in FZA, we were able to detect a within-subject difference in FZA of 8%.

### Statistics

Descriptive statistics (means ± SDs) were computed for each of the dependent variables at baseline and the end of MP1 and MP2. Pearson correlations were computed between PZCs and variables of whole-body zinc homeostasis (i.e., EZP, FZA, and TAZ), total and oxidized markers of DNA damage, and serum proteins associated with DNA damage, oxidative stress, and inflammation. Differences between MP1 and MP2 were determined for all endpoints with the use of linear models with generalized estimating equations to account for repeated measures of a subject. Pairwise significant differences were assessed with the use of Tukey’s adjustment for multiple comparisons. Statistical analyses were conducted with SAS version 9.4 software (SAS Institute), and *P* = 0.05 was used as the cutoff for statistical significance except for differences in serum proteins between MP1 and MP2 when *P* ≤ 0.01 was used to account for the number of multiple comparisons. This *P* value was arbitrarily chosen for multiple comparisons (*n* = 1129) between serum proteins because it was more conservative and appropriate for the large number of variables analyzed.

## RESULTS

To determine the effects of modest increases in dietary zinc on measures of zinc status and metabolic functions, we conducted a 6-wk consumption study in which participants were provided 3 meals/d with controlled amounts of zinc. Eighteen healthy men participated in the study (**Table 1**). The ethnicity of participants was diverse and reflected an attempt to recruit participants who were accustomed to eating a rice-based diet. The initial mean BMI and mean ± SD total body weight of participants were 22 and 68 ± 5 kg, respectively, with 16% as fat. The mean ± SD prestudy dietary zinc intake from 10 of the men was 13.0 ± 6.5 mg/d, which provided 1980 ± 912 kcal/d. The mean ± SD initial PZCs averaged 75 ± 10 μg/dL; none of the men had initial values <60 μg/dL. Blood hemoglobin concentrations were normal in all of the men. None of the measured hematologic variables or other health indicators changed significantly during the study (**Supplemental Table 3**).

**TABLE 1:** Participant characteristics

	Value
Ethnicity, %	
Caucasian	33
Asian	28
Indian	17
Other	22
Age, y	23 ± 4 (20–37)^1^
Height, m	1.75 ± 0.05 (1.69–1.85)
Weight, kg	67.8 ± 5.4 (58.7–77.6)
BMI, kg/m^2^	22 ± 2 (19–25)
Body composition,^2^ %	
Lean mass	84 ± 4 (76–92)
Fat mass	16 ± 4 (8–24)
Prestudy dietary zinc,^3^ mg/d	13.4 ± 6.6 (5.7–28.1)
Plasma zinc,^4^ μg/dL	75.0 ± 10.2 (61.4–102.5)
Hemoglobin,^5^ g/dL	15.6 ± 0.7 (14.6–16.8)

1 Mean ± SD; range in parentheses (all such values).

2 Measured with the use of a BOD POD whole-body air-displacement plethysmography device (COSMED USA).

3 Data from 10 men.

4 Baseline plasma zinc was measured with the use of inductively coupled plasma optical emission spectrophotometry.

5 Baseline hemoglobin was measured with the use of an automated complete blood count instrument (ADVIA 120 Hematology System; Siemens Healthcare GmbH).

The study diet, with protein sources that were limited to egg whites, fish, and chicken breasts, provided 6.4 ± 1.5 mg Zn/d (**Table 2**). The mean total phytate content of the study diet was 0.7 g/d. In MP1, when 1.5 g exogenous phytate was added to the diet, mean phytate intake was 2.2 g/d, and the phytate:zinc molar ratio was 35.1. PZCs did not change during the study (Table 2). The size of the EZP tended to increase by ∼15% during MP2 (*P* = 0.058). Because the EZP is located within the fat-free mass, the size of the EZP per kilogram of fat-free mass was calculated, and it did not differ between MP1 and MP2. The FZA decreased significantly from 35% to 28% between MP1 and MP2, respectively (*P* < 0.005), but TAZ increased from 2.1 to 2.8 mg/d, respectively (*P* < 0.005).

**TABLE 2:** Study dietary zinc and phytate amounts and measures of plasma zinc, EZP size, and fractional and total zinc absorption^1^

Metabolic period	Low zinc, MP1	Moderate zinc, MP2	*P*^2^
Dietary zinc,^3^ mg/d	6.37 ± 1.54^4^	10.37^5^	—
Phytate, g/d	2.2	0.7	—
Phytate:zinc molar ratio	35.1	7.1	—
Plasma zinc, μg/dL	74.0 ± 10.1	78.8 ± 14.5	NS
EZP,^6^ mg	136 ± 43	154 ± 28^3^	0.058
EZP, mg/kg FFM	2.64 ± 0.67	2.31 ± 0.73	NS
FZA, %	34.6 ± 13.8	28.2 ± 11.5^6^	0.005
Total absorbed zinc,^7^ mg/d	2.1 ± 0.9	2.8 ± 1.2^6^	0.005

1 EZP, exchangeable zinc pool; FFM, fat-free mass; FZA, fractional zinc absorption; MP1, metabolic period 1; MP2, metabolic period 2.

2 Paired *t* tests were used to calculate the significance between MP1 and MP2.

3 Dietary zinc was increased in MP2 by adding 1.33 mg Zn from zinc sulfate to each of the 2 meals for a total of an additional 4 mg Zn/d.

4 Mean ± SD (all such values).

5 Mean (all such values).

6 Stable-isotope studies for measuring the EZP at the end of the low-zinc period were measured with the use of ^70^Zn during the first week of MP2, and the EZP for the end of the moderate zinc period was measured the week after the completion of MP2. *n* = 15.

7 Total absorbed zinc equals the FZA (percentage) × dietary zinc (milligrams per day). The FZA for MP1 and MP2 was measured on day 4 of each period. *n* = 18.

In addition to whole-body homeostatic biomarkers of zinc status, we measured the blood cell zinc content and the expression of some leukocytic zinc transporters (**Table 3**). The 4-mg increase in dietary zinc between MP1 and MP2 did not increase erythrocyte or leukocytic zinc concentrations. Relative to the baseline, the gene expression for the zinc importers (Zip1, Zip4, and Zip8) and the zinc exporters (ZnT7 and ZnT1) did not significantly change over the course of the study. Metallothionein (Mt2a) gene expression also did not change.

**TABLE 3:** Cellular measures of zinc status^1^

Indicator	Baseline	Low zinc, MP1	Moderate zinc, MP2
Erythrocyte zinc, fg/10^6^ cells	20.84 ± 2.46^2^	20.18 ± 2.06	20.13 ± 2.10
Leukocyte zinc, fg/cell	10.22 ± 2.95	10.38 ± 2.55	10.83 ± 2.40
Gene expression, fold change from baseline^3^			
Zip1	1.0^4^	0.92 ± 0.54	1.12 ± 0.55
Zip4	1.0	0.98 ± 0.29	1.11 ± 0.57
Zip8	1.0	0.84 ± 0.39	0.85 ± 0.37
ZnT1	1.0	1.39 ± 0.47	1.27 ± 0.62
ZnT7	1.0	1.42 ± 0.71	1.35 ± 0.58
Mt2a	1.0	1.15 ± 0.58	1.24 ± 1.29

1 There were no significant differences in any cellular measures of zinc status. Baseline values were measured on day 1, MP1 values were measured on day 15, and MP2 values were measured on day 43. MP1, metabolic period 1; MP2, metabolic period 2; Mt2a, metallothionein 2a.

2 Mean ± SD (all such values).

3 Relative messenger RNA concentrations of zinc transporters and Mt2a at the end of MP1 and MP2 are expressed as ratios to baseline values with all baseline data normalized to 1.0. All mRNA concentrations are expressed relative to housekeeping genes as described in Methods.

4 Mean (all such values).

**FIGURE 1:**
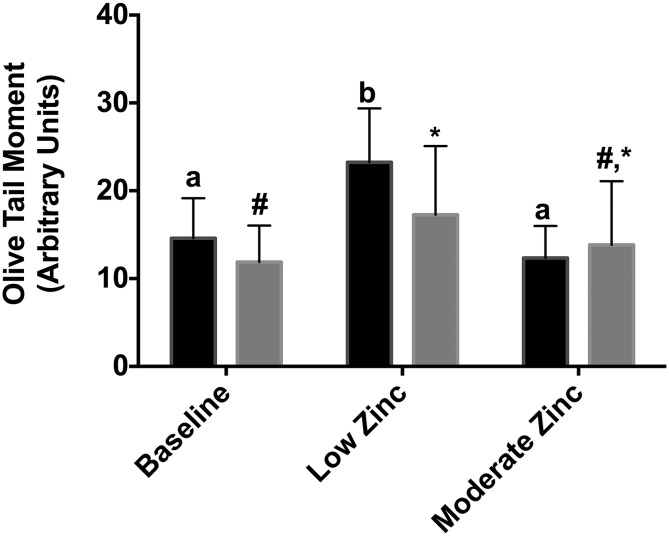
Mean ± SD DNA strand breaks and oxidized lesions measured with the use of the alkaline comet and FPG comet assays. Olive tail moments (arbitrary units) from the alkaline comet assay are depicted with black bars; tail moments from FPG comets are depicted with gray bars. *n* = 18 for alkaline comets, and *n* = 13 for FPG comets. Significant differences between means were determined with the use of a repeated-measures ANOVA followed by Bonferroni’s multiple-comparisons test. Bars with the same letter or symbol represent means that are not significantly different from each other. DNA strand breaks increased from baseline (14.5% ± 4.7%) to the end of the low-zinc period (23.0% ± 6.3%; *P* < 0.001) and decreased by the end of the higher-zinc period (12.2% ± 3.7%; *P* < 0.001). Olive tail moments that were due to oxidation increased significantly from baseline (11.88% ± 4.17%) to the end of the low-zinc period (17.26% ± 7.83%; *P* = 0.01). FPG, formamidopyrimidine DNA-glycosylase.

DNA single-strand breaks were measured as a cellular zinc functional biomarker at baseline and at the end of the low and moderate zinc metabolic periods (**Figure 1**). DNA damage, as indicated by the olive tail moment, increased from baseline (14.5% ± 4.7%) to the end of the low-zinc period (MP1) (23.0% ± 6.3%; *P* < 0.001) and decreased by the end of the higher-zinc period (MP2) (12.2% ± 3.7%; *P* < 0.001). DNA strand breaks can occur as a result of either DNA damage or repair. We also measured the number of strand breaks that were generated in response to the addition of FPG, which is an enzyme that removes oxidized purines. Compared with baseline, the number of FPG-sensitive strand breaks that resulted from oxidized purines increased significantly with the low-zinc diet (MP1) (Figure 1). However, at the end of MP2, the number of DNA strand breaks that were generated by oxidative DNA damage was not significantly different from that at the end of MP1 or at baseline. Thus, the additional 4 mg Zn/d that was consumed for 4 wk reduced the overall DNA damage, but it did not fully promote the repair of oxidized lesions.

To assess the response of serum proteins that are involved in DNA repair, oxidative stress, or inflammation with the response to a 4-mg change in dietary zinc, we measured the concentrations of 1129 serum proteins with the use of the SOMAscan proteomic assay. The statistical analysis, with the use of paired *t* tests, showed that 116 proteins changed significantly (*P* < 0.01) between the 2 metabolic periods (**Supplemental Table 4**). With the addition of 4 mg dietary zinc, the amounts of 61 proteins decreased, of which the majority were proinflammatory in nature. The amounts of 55 proteins increased, most of which were involved in an adaptive immune function or cellular metabolism.

To better understand the metabolic basis of the DNA strand breaks, we identified the proteins that are involved in the following 3 key areas where zinc function maybe implicated: *1*) DNA damage and repair, *2*) oxidative stress, and *3*) the response to inflammation (**Table 4**). Proteins that are involved in the inflammatory response were analyzed because it has been shown that inflammation is secondary to DNA damage and oxidative stress (27, 28). For example, of the 5 DNA-damage proteins that we identified, 2 repair proteins [high-mobility group protein B1 (HMGB1) and ubiquitin-conjugating enzyme E2N] increased significantly with the addition of 4 mg dietary zinc. Concomitantly, proteins reflecting DNA strand breaks [TNF receptor superfamily member 1B, importin subunit α-1, and E3 ubiquitin-protein ligase (Mdm2)] decreased during the same time period. In category 2 (oxidative stress), plasma antioxidants (i.e., peroxiredoxin-1, ferritin, and catalase) increased with the added dietary zinc, whereas proteins that are involved in ROS formation decreased (i.e., lactoperoxidase). In category 3 (inflammation), anti-inflammatory proteins increased (i.e., apolipoprotein A-I, HMGB1, and IL-2 receptor subunit α), whereas proteins that are involved in the response to inflammation decreased (i.e., fibrinogen, resistin, and fibronectin fragment 4) with the additional dietary zinc. Taken together, these results show that metabolic functions that are associated with maintaining DNA integrity improved with the addition of a small amount of dietary zinc despite an absence in the response of traditional zinc-status biomarkers.

**TABLE 4:** Plasma proteins responding to a moderate 4 mg increase in dietary zinc^1^

	MP1-to-MP2 change	*P*
Category 1: DNA damage and repair, %		
TNF receptor superfamily member 1B	−7.62 ± 1.41	0.0002
Importin subunit α-1	−6.09 ± 0.04	0.001
E3 ubiquitin-protein ligase Mdm2	−4.08 ± 0.02	0.003
High mobility group protein B1	21.89 ± 2.82	0.001
Ubiquitin-conjugating enzyme E2 N	13.91 ± 1.34	0.01
Category 2: oxidative stress, %		
MHC class I polypeptide-related sequence B	−8.7 ± 0.14	0.0002
Platelet-derived growth factor receptor β	6.71 ± 2.19	0.01
Lactoperoxidase	−8.01 ± 0.3	0.01
Peroxiredoxin-1	17.33 ± 0.76	0.001
6-phosphogluconate dehydrogenase, decarboxylating	19.51 ± 1.99	0.001
Ferritin	24.95 ± 0.52	0.005
Catalase	13.95 ± 7.14	0.01
Prostaglandin G/H synthase 2	6.18 ± 0.11	0.01
Aflatoxin B1 aldehyde reductase member 2	13.52 ± 0.29	0.01
Category 3: inflammation, %		
TNF receptor superfamily member 1B	−7.62 ± 1.41	0.0002
Fibrinogen	−5.54 ± 2.03	0.003
Inter-α-trypsin inhibitor heavy chain H4	−6.33 ± 4.09	0.003
Resistin	−5.98 ± 0.18	0.004
C-C motif chemokine 4-like	−8.73 ± 0.1	0.01
TNF ligand superfamily member 6, soluble form	−6.05 ± 0.12	0.01
α-1-Antichymotrypsin	−7.7 ± 5.84	0.01
Fibronectin fragment 4	6.32 ± 1.57	0.01
Apolipoprotein A-I	10 ± 1.87	0.0004
High mobility group protein B1	21.89 ± 2.82	0.001
Ribosomal protein S6 kinase α-5	9.13 ± 0.04	0.003
IL-2 receptor subunit α	3.07 ± 0.07	0.005

1 All values are means + SEMs. *n* = 18. Paired *t* tests were used to determine significance. *P* < 0.01 was used as an arbitrary cutoff for significance. MHC, major histocompatibility complex; MP1, metabolic period 1; MP2, metabolic period 2.

To determine whether these metabolic bioindicators are more sensitive than traditional zinc biomarkers to changes in dietary zinc, we correlated the changes in PZCs and EZP sizes with changes in the previously detailed proteins between MP1 and MP2. Plasma zinc and the size of the EZP did not correlate significantly with any of the responsive proteins (data not shown). However, the changes in oxidative DNA damage, as measured by FPG comets, which occurred with the dietary-zinc change, were correlated significantly with changes in HMGB1 (*r* = 0.896, *P* = 0.0002), which is a marker of inflammation that is specifically involved in endotoxin binding to lipopolysaccharide-binding protein and to prostaglandin G/H synthase 2 (*r* = 0.82, *P* = 0.02), which is a marker of peroxidase activity. These data suggest that DNA strand breaks (both total and oxidized) are sensitive biomarkers of a change in metabolic wellbeing in response to dietary zinc.

## DISCUSSION

A short-term moderate increase in dietary zinc from 6 to 10 mg/d caused a reduction in leukocyte DNA damage and significant changes in concentrations of serum proteins that are associated with DNA damage, oxidative stress, and inflammation. These shifts in dietary zinc–related functions occurred without a change in plasma, erythrocyte, or leukocyte zinc concentrations or the EZP size. Also, the gene expression for 5 key zinc transporters and for metallothionein 2a in leukocytes did not respond to the modest increase in dietary zinc. Previous reports of changes in the gene expression of zinc transporters or metallothionein occurred with larger changes in dietary zinc (29–31).

The comet assay has been used previously in several human-intervention trials to measure DNA single-strand breaks from peripheral blood cells (12, 23, 32). In a previous zinc depletion–zinc repletion study by our group, DNA damage increased significantly in healthy men after the consumption of a diet that provided 4 mg Zn/d for 6 wk, including a formula diet that provided 0.6 mg Zn/d during the first week. Repletion with 11 mg Zn/d for 4 wk with a 20-mg Zn supplement for the first 7 d ameliorated the DNA damage. Although PZCs did not change significantly during zinc depletion in that study, there was a negative correlation between PZCs and DNA damage in the 9 men. DNA strand breaks also declined significantly in a group of Ethiopian women who were given 20 mg supplemental zinc/d for 17 d (32). This change occurred without an overall increase in PZCs, but PZCs and DNA strand breaks were negatively correlated at the end of the study. Intracellular DNA strand breaks, which are a potential functional indicator of zinc status, appeared to be sensitive to small changes in dietary zinc, which suggested that DNA integrity is one of the first metabolic functions to improve with modest increases in zinc intake.

In the proteomic analysis, 26 proteins that are involved with DNA damage, oxidative stress, and inflammation responded to an increase of 4 mg dietary zinc (Table 4). For example, the amount of proteins that are associated with DNA repair was greater at the end of MP2 (i.e., HMGB1 and ubiquitin-conjugating enzyme E2N). HMGB1 maintains the integrity of DNA by several mechanisms including participating in nucleotide excision repair, mismatch repair, base-excision repair, and double-strand break repair (33). In addition, HMGB1 has been postulated to be a redox and inflammation sensor (34).

Zinc may also prevent DNA damage by protecting cellular components from oxidation. Although zinc is a redox inert metal and does not participate in oxidation-reduction reactions (35), it can reduce oxidant stress by protecting protein sulfhydryl groups from oxidation by antagonizing redox-active transition metals such as iron or copper, thereby preventing their oxidation of protein sulfhydryl groups (36). An example of this function was the significant increase in ferritin when dietary zinc was increased (Table 4). Ferritin reduces protein oxidation by binding and converting redox-active iron to a nontoxic form. The role of zinc in protein folding and enabling the proper functioning of proteins may explain how an increase in peroxiredoxin-1, which is a cysteine-rich protein with both antioxidant and protein-folding chaperone functions, is linked to reduced oxidative stress. The increase in peroxiredoxin-1 with the dietary zinc increase may have enabled appropriate protein folding that facilitated its function as an antioxidant (37).

Zinc reduces oxidative stress and also limits the formation of inflammatory cytokines by upregulating the zinc-finger protein A20, which inhibits nuclear transcription factor κB activation (35). When dietary zinc was increased by 4 mg/d in this study, the proteins that are associated with an increase in inflammation (i.e., fibrinogen, resistin, and TNF ligand superfamily member 6, soluble form) declined, whereas the proteins that are linked to a decrease in inflammation increased (i.e., apolipoprotein A-I, HMGB1, and IL-2 receptor subunit α). To our knowledge, the relation between apolipoprotein A-I and plasma zinc has not been studied, but low zinc availability in the media of a human liver cancer cell line reduced apolipoprotein A-I mRNA (38). However, human as well as tissue-culture studies have shown that zinc is required for IL-2 receptor subunit α expression and for reducing the underlying inflammation that accompanies a decline in IL-2 receptor subunit α mRNA (39, 40).

Oxidative stress and chronic inflammation play causative roles in the development of chronic diseases including cancers, atherosclerosis, and other degenerative disorders. Our results suggest that subclinical zinc deficiencies may shift cellular metabolic functions in a manner that increases chronic disease risk. Epidemiologic studies have reported associations between zinc and risks of cancer, cardiovascular disease, and diabetes (41–45), but to our knowledge, this is the first human study to show that a moderate increase in dietary zinc alters underlying cellular changes that are associated with subsequent disease risk. Further studies are needed to show that a moderate increase in dietary zinc could positively influence biomarkers that are associated with these diseases.

To date, stunting and an increased risk of gastrointestinal or pulmonary infections are the 2 primary functional consequences of zinc deficiency in humans. On the basis of the current study and studies reported by other authors (12, 32), we suggest that DNA damage should be considered a functional indicator of human zinc deficiency. The use of comet assay as a functional indicator of zinc nutrition has several advantages over the assessment of incidences of stunting and morbidity. First, it can be used in studies of adults as well as children. Second, unlike stunting and increased morbidity, it is very sensitive to small changes in dietary zinc, and third, it requires very little blood, which makes it possible to do the analyses of a finger-stick sample that is drawn in a field setting. The association between DNA damage and dietary zinc appears to be stronger than that with PZCs. The same is true for stunting (46). Although PZCs respond readily to oral zinc supplements that are taken without food, plasma zinc does not decline with lower dietary zinc until intakes are <3 mg/d (47). Thus, these zinc-related functions appear to be more sensitive to changes in the dietary zinc supply than are PZCs. However, this DNA damage is not specific for low zinc intake. DNA strand breaks also increase with low intakes of folate and vitamin B-6 (48, 49).

The multiple metabolic responses to a marginal increase in dietary zinc that were observed in this study suggest that the framework that is used to evaluate zinc status needs to be modified. Modest changes in dietary zinc, as is typical with changes in the composition of human diets, caused a shift in the serum concentrations of multiple proteins, thereby reflecting several metabolic functions rather than a single, specific biomarker. Proteins that are associated with preventing DNA damage, oxidative stress, and inflammation changed without any changes in PZCs. We suggest that a combination of endpoints that are linked to a key metabolic function (i.e., DNA damage) can be used as bioindicators of the relation between zinc nutrition and a nonspecific functional outcome. For example, a shift in a few of the zinc-sensitive proteins that are involved in DNA damage (e.g., peroxiredoxin-1, ferritin, fibrinogen, and resistin) could be used to confirm that dietary zinc inadequacy contributes to the observed DNA lesions.

In conclusion, we show that a moderate increase in dietary zinc, which is similar to that when regular rice is replaced with zinc-biofortified rice, reduces DNA strand breaks and alters circulating concentrations of plasma proteins that are involved in DNA repair, oxidative stress, and inflammation. DNA damage is not a specific bioindicator of zinc nutrition. Nevertheless, DNA damage can serve as a bioindicator of zinc nutrition when measurements of zinc-sensitive proteins like peroxiredoxin-1, ferritin, fibrinogen, and resistin are also taken into consideration.

## Supplementary Material

Online Supporting Material

## Footnotes

5 Abbreviations used: EZP, exchangeable zinc pool; FPG, formamidopyrimidine DNA-glycosylase; FZA, fractional zinc absorption; HMGB1, high-mobility group protein B1; ICP-OES, inductively coupled plasma optical emission spectrophotometry; MP1, metabolic period 1; MP2, metabolic period 2; mRNA, messenger RNA; NIST, National Institute of Standards and Technology; PCR, polymerase chain reaction; ppm, parts per million; PZC, plasma zinc concentration; ROS, reactive oxygen species; TAZ, total absorbed zinc.
