# Supplementary material for: A moderate increase in dietary zinc reduces DNA strand breaks in leukocytes and alters plasma proteins without changing plasma zinc concentrations
Source: Am J Clin Nutr. 2016 Dec 21;105(2):343–51. doi: 10.3945/ajcn.116.135327 (PMC5267297; doi:10.3945/ajcn.116.135327)
Supplement: Online Supporting Material [file supp_105_2_343__index.html]

A moderate increase in dietary zinc reduces DNA strand breaks in leukocytes and alters plasma proteins without changing plasma zinc concentrations — Online Supporting Material 

# A moderate increase in dietary zinc reduces DNA strand breaks in leukocytes and alters plasma proteins without changing plasma zinc concentrations

## Online Supporting Material

- Online Supporting Material - Tables 1-4
