# Supplementary material for: A moderate increase in dietary zinc reduces DNA strand breaks in leukocytes and alters plasma proteins without changing plasma zinc concentrations
Source: Am J Clin Nutr. 2016 Dec 21;105(2):343–51. doi: 10.3945/ajcn.116.135327 (PMC5267297; doi:10.3945/ajcn.116.135327)
Supplement: Online Supporting Material [file 116.135327_ajcn135327SupplementaryData1.docx]

**Supplemental Table 1.**

Study Diet Menus

|  | **Menu Day One** | **Menu Day Two** | **Menu Day Three** | **Menu Day Four** |
| --- | --- | --- | --- | --- |
| **Breakfast** | Curried Puffed Rice | Rice | Curried Puffed Rice | Rice |
|  |  | Curried Egg |  | Curried Egg |
|  |  |  |  |  |
| **Lunch** | Rice | Rice | Rice | Rice |
|  | Dal- Lentils | Dal- Lentils | Dal- Lentils | Dal- Lentils |
|  | Curried Egg | Potato, Cauliflower, & Tomato | Curried Egg | Mashed Potato |
|  | Potato & Spinach | Spiced Spinach | Cabbage & Potato | Eggplant & Tomato |
|  | Cauliflower & Peas |  | Eggplant & Spinach |  |
|  |  |  |  |  |
| **Dinner** | Kichuri- Curried Rice & Lentils | Rice | Rice | Rice |
|  | Curried Chicken | Dal- Lentils | Dal- Lentils | Dal- Lentils |
|  | Green Peppers | Curried Fish | Curried Chicken | Cauliflower & Pumpkin Curry |
|  |  | Buttered Cabbage | Ginger Cauliflower | Spinach |
|  |  | Eggplant & Green Peppers | Curried Green Peppers |  |
|  |  |  |  |  |
| **Snacks** | Tea- 1 Black Tea Bag | Tea- 1 Black Tea Bag | Tea- 1 Black Tea Bag | Tea- 1 Black Tea Bag |
|  | Lowfat Milk (1%)- 4 Tablespoons | Lowfat Milk- 8 Tablespoons | Lowfat Milk- 4 Tablespoons | Lowfat Milk- 6 Tablespoons |
|  | Sugar- 6 teaspoons | Sugar- 4 teaspoons | Sugar- 4 teaspoons | Sugar- 4 teaspoons |
|  | Rice Beverage^1^- 2 cups | Cucumber- 60g | Orange | Cucumber- 60g |
|  | Banana |  | Cucumber- 60g |  |
|  | Cucumber- 60g |  |  |  |

^1^Variable amounts of a rice beverage or lemonade were given to the subjects to adjust energy intakes to maintain body weight. These supplements were provided 80% of the energy from carbohydrate, 10% from protein and 10% from fat. The rice beverage contained egg whites, sugar, rice flour, and rice flakes. The lemonade contained lemonade concentrate, maltodextrin, egg white, and corn oil.

**Supplemental Table 2.**

Primers for RT-PCR of zinc transporter genes and metallothionein

| **Gene** | **Forward primer (5’-3’)** | **Reverse primer (5’-3’)** |
| --- | --- | --- |
| **Zip1** | GATTGGGGAAGACACTTGACTGCT | GAAAGAGGAAGGGGATTTGTTTGG |
| **Zip4** | AGACTGAGCCCAGAGTTGAGGCTA | TGTCGCAGAGTGCTACGTAGAGGA |
| **Zip8** | CCTCGGATTGATTTTGACTCCACT | AGCAGGATTTGCATAGCATGTCAC |
| **Znt1** | GCATCAGTTTATGAGGCTGGTCCT | CAGGCTGAATGGTAGTAGCGTGAA |
| **Znt7** | TTTCTTCCTGTGCCTGAACCTCTC | GAGTCGGAAATCAAGCCTAAGCAG |
| **Mt2a** | TGCATTTGCACTCTTTGCAT | CTTCAGCTCGCCATGGAT |
| **18S** | GCAATTATTCCCCATGAACG | GGGACTTAATCAACGCAAGC |
| **β-actin** | CGCCCAGGCACCAGGGC | GCTGGGGTGTTGAAGGT |

**Supplemental Table 3.**

Hematologic and white blood cell variables for various study days during the metabolic periods^1^.

|  | **Study day** |  |  |
| --- | --- | --- | --- |
|  | 1 | 15 | 43 |
| **WBC parameters** |  |  |  |
| total WBC, x10^6/ml | 5.83 ± 1.18 | 5.25 ± 1.29 | 5.52 ± 1.33 |
| neutrophil, % | 52 ± 10 | 52 ± 10 | 52 ± 9 |
| lymphocyte, % | 33 ± 5 | 35 ± 9 | 35 ± 8 |
| monocyte, % | 7 ± 1 | 7 ± 1 | 7 ± 2 |
|  |  |  |  |
| **RBC parameters** |  |  |  |
| total RBC, x10^9/ml | 5.37 ± 0.07 | 5.15 ± 0.07 | 5.25 ± 0.08 |
| MCV, fL | 85.69 ± 4.55 | 87.02 ± 4.23 | 86.87 ± 3.81 |
| MCH, pg | 29.09 ± 1.70 | 29.76 ± 2.08 | 29.25 ± 1.50 |
| MCHC, g/dL | 34.21 ± 1.32 | 34.28 ± 1.53 | 33.67 ± 1.05 |
| RDW, % | 12 ± 1 | 12 ± 1 | 13 ± 1 |
|  |  |  |  |
| **PLT parameters** |  |  |  |
| total PLT, x10^6/ml | 232.07 ± 44.89 | 207.22 ± 56.98 | 211.61 ± 51.40 |
| MPV, fL | 8.37 ± 1.66 | 9.18 ± 1.37 | 9.67 ± 0.97 |
| PCT, % | 0.21 ± 0.07 | 0.20 ± 0.05 | 0.20 ± 0.05 |
| PDW, % | 52 ± 9 | 52 ± 5 | 52 ± 5 |

^1^Hematological values are shown as means ± SD, *n* = 13-18 per group, as a function of study day. No significant differences were observed in any parameter for any study day, compared to control.

Abbreviations are as follows: mean corpuscular hemoglobin (MCH), mean corpuscular hemoglobin concentration (MCHC), mean corpuscular volume (MCV), mean platelet volume (MPV), platelet (PLT), platelet blood cell distribution width (PDW), plateletcrit (PCT), red blood cell (RBC), red blood cell distribution width (RDW), and white blood cell (WBC).

**Supplemental Table 4.**

Effect of a Moderate 4 mg Increase in Diet Zinc on Serum Proteins^1,2^

| **Proteins with increased expression** | **p-value** | **Uniprot ID** |
| --- | --- | --- |
| Apolipoprotein A-I | 0.0004 | P02647 |
| High mobility group protein B1 | 0.001 | P09429 |
| Interleukin-3 | 0.001 | P08700 |
| Fibroblast growth factor 16 | 0.001 | O43320 |
| Stress-induced-phosphoprotein 1 | 0.001 | P31948 |
| 6-phosphogluconate dehydrogenase, decarboxylating | 0.001 | P52209 |
| Desert hedgehog protein N-product | 0.001 | O43323 |
| Peroxiredoxin-1 | 0.001 | Q06830 |
| S-phase kinase-associated protein 1 | 0.002 | P63208 |
| Pyridoxal phosphate phosphatase | 0.002 | Q96GD0 |
| Amphiregulin | 0.002 | P15514 |
| Ribosomal protein S6 kinase alpha-5 | 0.003 | RPS6KA5 |
| Junctional adhesion molecule C | 0.003 | Q9Y624 |
| Dynactin subunit 2 | 0.003 | Q13561 |
| Fetuin-B | 0.004 | Q9UGM5 |
| Protein kinase C iota type | 0.004 | PK1743 |
| NKG2D ligand 3 | 0.004 | Q9BZM4 |
| Ubiquitin-conjugating enzyme E2 L3 | 0.004 | P68036 |
| Proteasome subunit alpha type-6 | 0.004 | P60900 |
| Copine-1 | 0.004 | Q99829 |
| Breast cancer anti-estrogen resistance protein 3 | 0.004 | P56945 |
| Ferritin | 0.005 | P02794 |
| Hepatocyte growth factor activator | 0.005 | Q04756 |
| Interleukin-2 receptor subunit alpha | 0.005 | P01589 |
| Cadherin-15 | 0.01 | P55291 |
| Protein 4.1 | 0.01 | P11171 |
| Ras-related C3 botulinum toxin substrate 1 | 0.01 | P63000 |
| Apolipoprotein E | 0.01 | P02649 |
| Inosine-5'-monophosphate dehydrogenase 2 | 0.01 | P12268 |
| Prostaglandin G/H synthase 2 | 0.01 | P35354 |
| DnaJ homolog subfamily B member 1 | 0.01 | P25685 |
| Serine/threonine-protein kinase 17B | 0.01 | O94768 |
| Phosphatidylethanolamine-binding protein 1 | 0.01 | P30086 |
| AT-rich interactive domain-containing protein 3A | 0.01 | Q99856 |
| Interferon gamma | 0.01 | P01579 |
| Methionine aminopeptidase 2 | 0.01 | P50579 |
| Growth hormone receptor | 0.01 | P10912 |
| Aflatoxin B1 aldehyde reductase member 2 | 0.01 | Q43488 |
| Small glutamine-rich tetratricopeptide repeat-containing protein alpha | 0.01 | O43765 |
| Casein kinase II 2-alpha:2-beta heterotetramer | 0.01 | P68400 |
| Alpha-soluble NSF attachment protein | 0.01 | P54920 |
| DNA topoisomerase 1 | 0.01 | P11387 |
| Mitochondrial import inner membrane translocase subunit TIM14 | 0.01 | Q96DA6 |
| Low affinity immunoglobulin gamma Fc region receptor III-B | 0.01 | O75015 |
| Beta-Ala-His dipeptidase | 0.01 | Q96KN2 |
| Heterogeneous nuclear ribonucleoprotein A/B | 0.01 | P22626 |
| Catalase | 0.01 | P04040 |
| Macrophage scavenger receptor types I and II | 0.01 | P21757 |
| Ubiquitin-conjugating enzyme E2 N | 0.01 | P61088 |
| Neutral ceramidase | 0.01 | Q9NR71 |
| OCIA domain-containing protein 1 | 0.01 | Q9NX40 |
| Estradiol 17-beta-dehydrogenase 1 | 0.01 | P14061 |
| Inhibitor of growth protein 1 | 0.01 | Q9UK53 |
| Heterogeneous nuclear ribonucleoproteins A2/B1 | 0.01 | P22626 |
| Tumor necrosis factor receptor superfamily member 13C | 0.01 | Q96RJ3 |
| Glyceraldehyde-3-phosphate dehydrogenase | 0.01 | P04406 |
| Kinesin-like protein KIF23 | 0.01 | Q02241 |
|  |  |  |
| **Proteins with decreased expression** | **p-value** | **Uniprot ID** |
| Epiregulin | 0.0002 | O14944 |
| Lysozyme C | 0.0002 | P61626 |
| MHC class I polypeptide-related sequence B | 0.0002 | Q29980 |
| Tumor necrosis factor receptor superfamily member 1B | 0.0002 | P20333 |
| Tyrosine-protein kinase JAK2 | 0.0002 | O60674 |
| Delta-like protein 1 | 0.001 | O00548 |
| Leukocyte immunoglobulin-like receptor subfamily B member 2 | 0.001 | Q8N423 |
| Importin subunit alpha-1 | 0.001 | P52292 |
| Asialoglycoprotein receptor 1 | 0.001 | P07306 |
| Trypsin-3 | 0.001 | P35030 |
| Fc receptor-like protein 3 | 0.002 | Q96P31 |
| Granzyme A | 0.002 | P12544 |
| Persephin | 0.002 | O60542 |
| E3 ubiquitin-protein ligase Mdm2 | 0.003 | Q00987 |
| Fibrinogen | 0.003 | P02671 |
| Inter-alpha-trypsin inhibitor heavy chain H4 | 0.003 | Q14624 |
| Endoplasmic reticulum aminopeptidase 1 | 0.003 | Q9NZ08 |
| T-cell surface glycoprotein CD4 | 0.003 | P01730 |
| Resistin | 0.004 | Q9HD89 |
| Macrophage-capping protein | 0.004 | P40121 |
| Complement c4b | 0.004 | P0C0L5 |
| Neuropilin-1 | 0.004 | O14786 |
| Seprase | 0.005 | Q12884 |
| Plasma protease C1 inhibitor | 0.01 | P05155 |
| Stanniocalcin-1 | 0.01 | P52823 |
| Macrophage metalloelastase | 0.01 | P39900 |
| C-C motif chemokine 19 | 0.01 | Q99731 |
| C-C motif chemokine 4-like | 0.01 | Q8NHW4 |
| Complement component C9 | 0.01 | P02748 |
| Complement C1r subcomponent | 0.01 | P00736 |
| Lymphocyte activation gene 3 protein | 0.01 | P18627 |
| Lactoperoxidase | 0.01 | P22079 |
| Cytokine receptor-like factor 1:Cardiotrophin-like cytokine factor 1 Complex | 0.01 | No ID |
| Ciliary Neurotrophic Factor | 0.01 | P26992 |
| Alpha-(1,3)-fucosyltransferase 5 | 0.01 | Q11130 |
| Granulysin | 0.01 | P22749 |
| Natural cytotoxicity triggering receptor 1 | 0.01 | O76036 |
| Tumor necrosis factor receptor superfamily member 17 | 0.01 | Q02223 |
| Vascular endothelial growth factor receptor 3 | 0.01 | P35916 |
| Ficolin-2 | 0.01 | Q15485 |
| Integrin alpha-V: beta-5 complex | 0.01 | P06756 |
| Nidogen-1 | 0.01 | P10493 |
| Alpha-1-antichymotrypsin | 0.01 | P01011 |
| Iduronate 2-sulfatase | 0.01 | P22304 |
| Neurexophilin-1 | 0.01 | P58417 |
| Agouti-related protein | 0.01 | O00253 |
| Sialic acid-binding Ig-like lectin 7 | 0.01 | Q9Y286 |
| Tumor necrosis factor ligand superfamily member 6, soluble form | 0.01 | P48023 |
| Semaphorin-6A | 0.01 | Q9H2E6 |
| Granulins | 0.01 | P28799 |
| Ephrin-A4 | 0.01 | P52798 |
| Neurogenic locus notch homolog protein 1 | 0.01 | P46531 |
| SLAM family member 6 | 0.01 | Q96DU3 |
| Sialic acid-binding Ig-like lectin 14 | 0.01 | Q08ET2 |
| Platelet-derived growth factor receptor beta | 0.01 | P09619 |
| Fibronectin Fragment 4 | 0.01 | P02751 |
| Cathepsin H | 0.01 | P09668 |
| Coactosin-like protein | 0.01 | Q14019 |

^1^ n=18 for all proteins.

^2^ Paired t-tests were used to determine statistical significance.
